# Draft Genome Sequence of *Bacillus thuringiensis* subsp. *aizawai* HD133

**DOI:** 10.1128/genomeA.00909-17

**Published:** 2017-09-14

**Authors:** Zujiao Fu, Rong Xiao, Rongjun Luo, Zhan Hu, Hua Yang, Zhaohui Guo, Ping Lei, Shiping Shan

**Affiliations:** Hunan Institute of Microbiology, Changsha, People’s Republic of China

## Abstract

We report here the 6,512,057-bp draft genome sequence of *Bacillus thuringiensis* subsp.* aizawai* HD133. This strain contains at least 6 *cry* genes and 13 candidate biosynthetic gene clusters.

## GENOME ANNOUNCEMENT

*Bacillus thuringiensis* is a Gram-positive bacterium widely used for insect control in agriculture and forestry, the use of which is attributed largely to the parasporal crystals produced during sporulation. In recent years, numerous genomes of *B. thuringiensis* were sequenced, which provide a large amount of data, especially for new insecticidal crystal protein genes (1–3).

*B. thuringiensis* subsp. *aizawai* HD133 was isolated from *Plodia interpunctella* in England by H. T. Dulmage (4). Six different insecticidal crystal protein genes, including *cry1Aa*, *cry1Ab*, *cry1C*, *cry1D*, *cry1I*, and *cry2B* genes, and three insecticidal crystal proteins, including Cry1Ab, Cry1C, and Cry1D, have been detected in this strain (5). The abundant insecticidal crystal proteins attribute strain HD133 a higher insecticidal activity against *Mamestra configurata* and resistant *P. interpunctella* than *B. thuringiensis* subsp. *kurstaki* HD1 (4, 6).

In this study, genomic DNA from HD133 was used to construct an Illumina paired-end (PE) library. It was sequenced by a 420-bp run on an Illumina MiSeq platform, generating 93,848 clean-data single reads, totaling 2,085 Mbp, which were assembled in 319 contigs arranged in 289 scaffolds, and 210 scaffolds longer than 1,000 bp by SOAP*denovo* version 2.04 and GapCloser version 1.12, giving a consensus length of 6,512,057 bp at 320× coverage (largest scaffold, 367,518 bp; *N*_50_, 67,205 bp; *N*_90_, 15,157 bp). Forty-one scaffolds longer than 50,000 bp were compared to the GenBank nonredundant database limited to *B. thuringiensis* (taxid 1428) using BLASTN. Thirty-eight of them (totaling 3,852,982 bp) cover 96% of the complete genome of *B. thuringiensis* strain YWC2-8 (7), with 99% identity. Scaffold 36 (53,723 bp) and scaffold 41 (50,761 bp) cover 32% and 84% of plasmids YWC2-8-1 and YWC2-8-2 from strain YWC2-8, respectively. The average G+C content of all the scaffolds from HD133 is 34.74%.

Genome annotation was added by the NCBI Prokaryotic Genome Annotation Pipeline (http://www.ncbi.nlm.nih.gov/genomes/static/Pipeline.html) and the RAST server (8). tRNA and rRNA genes were identified by tRNAscan-SE version 1.3.1 (9) and Barrnap 0.4.2 (http://www.vicbioinformatics.com/software.barrnap.shtml), respectively. Annotation by Glimmer 3.02 predicted 6,671 coding sequences, with an average gene length of 784 bp. The whole genome contains 107 tRNA genes and 14 copies of 23S/5S and 16S rRNA genes.

The BtToxin_Scanner tool (10) was used to find insecticidal toxin genes present in the HD133 genome; 7 candidate *cry* sequences were detected in scaffolds 8, 36, 92, and 143. Among the 7 candidate *cry* sequences, 6 of them showed 100% identity to known *cry* genes, including *cry1Aa*, *cry1Ia*,* cry1Ca*,* cry1Da*,* cry2Ab*, and *cry9Ea*. The *cry1Ab* gene present in HD133 (4, 11) was not detected in this study, probably due to the loss during genomic DNA extraction or library construction, but the *cry2Ab* and *cry9Ea* genes were first detected from the strain.

The antiSMASH tool (http://antismash.secondarymetabolites.org/) was used to find the secondary metabolites in the HD133 genome, and 13 candidate secondary metabolite clusters were identified, including bacteriocins, nonribosomal peptide synthetases (Nrps), lantipeptide-Nrps-type 1 polyketide synthases (zwittermycin A biosynthetic gene cluster), terpenes, siderophores, sactipeptides, and other gene clusters.

### Accession number(s).

This whole-genome shotgun project has been deposited in DDBJ/ENA/GenBank under the accession no. NHZG00000000. The version described in this paper is version NHZG01000000.
